# The Breeder’s Gazette

**Published:** 1882-04

**Authors:** 


THE BREEDER'S GAZETTE. A weekly journal published at Chicago, devoted to the stock-breeder, turfman, and dairyman. Edited by Mr. J. H. Sanders, who ranks as one of the most eminent writers of the present day on live stock. The various departments are handled with force and vigor. It is published at $3 per annum.

